# The development of an evaluation framework for injury surveillance systems

**DOI:** 10.1186/1471-2458-9-260

**Published:** 2009-07-23

**Authors:** Rebecca J Mitchell, Ann M Williamson, Rod O'Connor

**Affiliations:** 1 NSW Injury Risk Management Research Centre, University of New South Wales, Sydney, Australia; 2 School of Public Health and Community Medicine, University of New South Wales, Sydney, Australia; 3 Department of Aviation, University of New South Wales, Sydney, Australia; 4 Rod O'Connor & Associates Pty Ltd, Sydney, Australia

## Abstract

**Background:**

Access to good quality information from injury surveillance is essential to develop and monitor injury prevention activities. To determine if information obtained from surveillance is of high quality, the limitations and strengths of a surveillance system are often examined. Guidelines have been developed to assist in evaluating certain types of surveillance systems. However, to date, no standard guidelines have been developed to specifically evaluate an injury surveillance system. The aim of this research is to develop a framework to guide the evaluation of injury surveillance systems.

**Methods:**

The development of an Evaluation Framework for Injury Surveillance Systems (EFISS) involved a four stage process. First, a literature review was conducted to identify an initial set of characteristics that were recognised as important and/or had been recommended to be assessed in an evaluation of a surveillance system. Second, this set of characteristics was assessed using SMART criteria. Third, those surviving were presented to an expert panel using a two round modified-Delphi study to gain an alternative perspective on characteristic definitions, practicality of assessment, and characteristic importance. Finally, a rating system was created for the EFISS characteristics.

**Results:**

The resulting EFISS consisted of 18 characteristics that assess three areas of an injury surveillance system – five characteristics assess data quality, nine characteristics assess the system's operation, and four characteristics assess the practical capability of an injury surveillance system. A rating system assesses the performance of each characteristic.

**Conclusion:**

The development of the EFISS builds upon existing evaluation guidelines for surveillance systems and provides a framework tailored to evaluate an injury surveillance system. Ultimately, information obtained through an evaluation of an injury data collection using the EFISS would be useful for agencies to recommend how a collection could be improved to increase its usefulness for injury surveillance and in the long-term injury prevention.

## Background

Planning for injury prevention and control activities relies upon good quality data from surveillance [1]. Most information on injuries is obtained from data collections that are intended for other purposes, such as hospital admission collections [2], which may not provide the core information needed for injury surveillance (i.e. what injuries occurred where to whom, when they occurred and why [3,4]). An assessment of a data collection is desirable to identify its capacity to perform injury surveillance and the likely accuracy and validity of conclusions that may be drawn from its data [5-7]. It is important to know the strengths and limitations of data collections as these define the limits for interpreting the analysis of the data in the collection.

Evaluation frameworks have been developed to assess public health [8,9], syndromic [10], and communicable disease [11] surveillance systems. However, these frameworks all recommend the assessment of a different selection of characteristics of a surveillance system, and all suggest varying definitions of, and methods to assess these characteristics. This lack of a standard approach for the assessment of characteristics makes it difficult to compare evaluation results across different surveillance systems. In addition, none of these frameworks provide details of how they were developed and why particular evaluation characteristics were included. The aim of this paper is to describe the development of a framework for the evaluation of an injury surveillance system. The availability of a clearly defined evaluation framework using agreed evaluation characteristics will both provide a sound and reproducible basis for analysis of the extent to which a data collection can be used for a particular purpose and for comparison of data collections.

## Methods

There were four main stages in the development of an evaluation framework for injury surveillance systems (EFISS). The first stage involved a review of the literature which identified the characteristics that have been used or recommended to be used to evaluate surveillance systems previously. The second stage reviewed these characteristics by testing them against a well-recognised set of criteria, the SMART criteria, and characteristics that did not meet this criteria were dropped. The third stage used expert judgments obtained using a modified-Delphi study to assess the remaining characteristics. Lastly, the final stage created a system for rating each characteristic. Ethics approval for the conduct of this research was obtained from the University of Queensland's Medical Research Ethics Committee.

### Stage 1 Identification of surveillance system characteristics

The aim of this stage was to review the literature to identify characteristics that had been used, or recommend to be used previously to evaluate a surveillance system. For this research, a characteristic was considered to be any attribute that might be assessed for a surveillance system. The review was undertaken using Medline (1960–2006), Embase (1982–2006), CINAHL (1960–2006), Web of Science (1960–2006), and Google™ using a variety of combinations of the key words related to the evaluation of surveillance systems 'surveillance', 'evaluation', 'guidelines', 'framework', 'injury surveillance', 'injury', 'comparison', 'review', 'assess', and 'quality'.

### Stage 2 Review of surveillance system characteristics

The aim of this stage was to review the characteristics identified from the literature by testing them against a well-recognised set of criteria. The characteristics were first categorised and then reviewed using SMART criteria (described below). The SMART criteria are based on goal-setting theory [12] and have been used in a wide range of settings to aid decision-making [13-15]. The SMART criteria were adapted so as to apply to evaluating characteristics of an injury surveillance system. Each characteristic was evaluated against the five criteria of the SMART framework, defined as:

• **S**pecific – the characteristic should be as detailed and specific as possible;

• **M**easurable – it should be possible to objectively assess or monitor the characteristic;

• **A**ppropriate – the characteristic should be suitable to assess an injury surveillance system and provide information central to injury surveillance;

• **R**eliable – the characteristic should be able to provide information that is consistent and reliable; and

• **T**ime-consistent – it should be possible to measure or monitor the characteristic consistently over time.

Each characteristic was reviewed using the SMART criteria by two authors (RM and AW). Characteristics where agreement was not initially reached were discussed and final SMART ratings for these characteristics were agreed upon. The characteristics that met all of the SMART criteria moved to the next stage of EFISS development.

### Stage 3: Assessment of characteristics by expert opinion

The aim of this stage was to use subject-matter experts in injury surveillance systems to provide expert opinion on the characteristics proposed to be included in the EFISS. Characteristics were tested in this stage using a two round modified-Delphi study [16,17]. A modified-Delphi study conducted using electronic-based questionnaires was selected (as opposed to in-person discussions) as experts were widely distributed around Australia. It also allowed panel members to provide their point of view anonymously and all opinions could be considered which is not always possible during in-person meetings where discussion can be dominated by a few individuals [18-20] or where opinions could be influenced by other individuals [19,21,22]. Moreover participants could complete the Delphi rounds at their leisure allowing more time for contemplation of responses [23].

Expert panel members were selected based on seven criteria: (i) working in the field of injury prevention; (ii) familiarity with the evaluation of surveillance systems; (iii) awareness of the strengths and limitations of surveillance systems; (iv) published on the evaluation of a surveillance system; (v) awareness of quantitative evaluation methods; (vi) familiarity with Australian injury data collections; and (vii) willingness to contribute. Fourteen panel members residing in Australia were identified from an examination of international and national conference proceedings and publications in the peer-review literature that they had either authored or co-authored on the evaluation of data collections. These people were contacted via email and invited to participate. All potential panel members worked in senior positions in injury research centres or public health research facilities. Seven ultimately participated, three epidemiologists and four public health professionals, a response rate of 50%. Of the non-participants, four did not reply to the original invitation, one declined due to excessive work commitments, one declined due to family reasons, and one initially agreed but later withdrew.

Panel members were given a generic user name and password and a unique link to an internet site to download a Microsoft^® ^Excel [24] file containing the questionnaires and background material for each modified-Delphi round. Each completed questionnaire was then uploaded to the internet site and the responses accessed and directly downloaded into SPSS [25] for analysis. Each questionnaire was pilot tested for content ambiguities on two individuals not familiar with the research.

Round one of the modified-Delphi focused on the subset of characteristics for which there was no consistent definition in the literature relevant to injury surveillance. The aim of this round was to reach consensus from a panel of experts on the suitability of 11 proposed characteristic definitions (ie. 6 data quality, 3 operational and 2 practical characteristics) for an injury surveillance system, the importance of these 11 characteristics for injury surveillance, and the practicality of assessing these 11 characteristics in an injury surveillance system. For this exercise, experts were asked to rate one definition, usually the most common from the review. However, all definitions of characteristics identified in the literature review were provided to the expert panel as background material. The panel were asked to rate: (i) the appropriateness of the proposed definitions of each characteristic; (ii) the practicality of assessing these characteristics; and (iii) the perceived importance of these characteristics for injury surveillance, and to suggest any modifications to proposed definitions.

A 5-point Likert scale, from '*not at all*' to '*extremely*', was used to rate each item. The expert panel was considered to have reached high consensus on an item when the proportion of all of the panel's ratings reached 70% and above, moderate consensus when the proportion of all ratings reached 50% to 69%, and low consensus if the proportion of all ratings was less than 50% [26].

A second round of the modified-Delphi had two purposes. Each panel member was asked to provide feedback on the appropriateness of the revised round one characteristic definitions and to rate the importance of all 28 characteristics for one of the three EFISS areas (i.e. data quality, operational, or practical characteristics of an injury surveillance system). For this round, the expert panel was provided with a summary of the panel's ratings and comments from round one, a summary of the revisions made to definitions following the panel's round one comments, and the revised definitions. The expert panel rated the appropriateness of the revised characteristic definitions using the same 5-point Likert scale as in the previous round. The panel rated the importance of all characteristics to assess either the data quality, operation, or practical capabilities of an injury surveillance system using a 7-point Likert scale from '*not at all*' to '*extremely*'. The 7-point Likert scale was selected to elicit more variability in responses, with the mean and median used to measure the central tendency of the distribution of the panel's ratings and the standard deviation (SD) and the interquartile range used to measure variability across the panel member's ratings.

Ratings were judged on the basis that the characteristic scored consistently high across raters. For a characteristic to be 'important' it was required to be judged by the majority of the panel as so, with a mean rating of 6.0 or higher adopted as a general cut-off to indicate a reasonably high level of importance. However, this meant that only data completeness, sensitivity, and representativeness would be included as data quality characteristics and specificity and positive predictive value (PPV) would be excluded. As both specificity and PPV were rated very close to the mean of 6.0 (i.e. 5.9) and both had high consensus (low SD), it was decided to consider both of these characteristics as also important for data quality. The SDs were adopted as a measure of consensus as a tight spread of scores indicated a high consensus (i.e. an SD between 0 and 1), a medium spread of scores inferred a moderate consensus (i.e. an SD greater than 1.0 and less than 2.0), and a wide spread of scores implied a weak or low consensus (i.e. an SD greater than 2.0) [26]. Specifically, a high score was judged as a mean rating of 5.9 and above and consistency was judged as high if the SD of ratings was one or less, moderate if it was between 1 and 2 SD's and low if it was more than 2 SD's. For inclusion in the EFISS, a characteristic needed both a high mean score and high consistency.

### Stage 4: Development of a rating system for the EFISS characteristics

The aim of this stage was to identify an appropriate rating system to use with the EFISS and to identify what would be considered to be both low and high ratings of each characteristic. Rating systems have been used in a wide range of areas to assist in the interpretation of assessment results, to facilitate comparison, and to obtain an overall rating of performance. A number of rating systems were investigated for possible application to the EFISS, including systems developed to rate the quality of scientific evidence [27,28], credit risk [29,30], tractor safety [31], professional sports [32], and vehicle safety [33,34]. In addition, the injury surveillance literature was reviewed to estimate what would be considered to be either *high *or *low *ratings for each characteristic.

## Results

### Stage 1 Identification of surveillance system characteristics

Twenty-four journal articles, book chapters, and reports were located that provided guidelines or made recommendations regarding characteristics that should be evaluated in a surveillance system. From these a list of 40 characteristics were identified (Table 1). These characteristics were: data completeness [4,8-10,35-37], sensitivity [4,8,9,35-47], specificity [4,41-43,47], representativeness [4,8-10,35-37,39-47], positive predictive value (PPV) [8,9,35-37,39-41,44-46], positive likelihood ratio (LR+) [48], clear purpose and objective(s) [8,10,35-37,40], data collection process [8-10,35-37,41], clear case definition [8,35,37,45,46,49], type of data collected is adequate for injury surveillance [8,38], use of uniform classification systems (i.e. standardised classification system) [4,8,10,36,45,46,49,50], system can be integrated with other data collections/compatible data collections [8,10,50], legislative requirement for collection of data [8-10,41,51], simplicity [4,8,9,35-37,39-46], timeliness [4,8-10,35-37,39-46,50,52], flexibility [4,8-10,35-37,39-46], quality control measures [36,37,52], data confidentiality [8-10,36,51,52], individual privacy [8-10,36,52], system security [8-10], stability of the system [8-10,41,47], data accessibility [4,10,47,49,50], acceptability [4,8-10,35-46], usefulness [6,8-10,35-37,40-44], data linkage potential [10,39,52], geocoding potential [10,39], compatible denominator data [36,37,39,49,50], routine data analysis [4,8,10,35-37,41,44-46], guidance material for data interpretation [36,41], routine dissemination of information [4,8,10,35-37,41,44-46,49,50,52], adequate resources/cost [8-10,36,38,40-44,49,50,52], communication support [41], coordination support [41], effectiveness of system in supporting programs [9,47], efficiency of resource use [9], portability [10], practicality of system [6,47], relevance of data to users [47], supervision support functions [41], and training support functions [41,50].

**Table 1:** Relevance of characteristics identified from the literature for inclusion within an evaluation framework for injury surveillance systems

**Characteristic^1^**	**Specific**	**Measurable**	**Appropriate**	**Reliable**	**Time-consistent**	**Meets SMART criteria**
**Data quality**						
Data completeness	Y	Y	Y	Y	Y	Y
Sensitivity	Y	Y	Y	Y	Y	Y
Specificity	Y	Y	Y	Y	Y	Y
Representativeness	Y	Y	Y	Y	Y	Y
Positive predictive value	Y	Y	Y	Y	Y	Y
Positive likelihood ratio	Y	Y	Y	Y	Y	Y
						
**Operational**						
Clear purpose and objective(s)	Y	Y	Y	Y	Y	Y
Data collection process	Y	Y	Y	Y	Y	Y
Clear case definition	Y	Y	Y	Y	Y	Y
Type of data collected is adequate for injury surveillance	Y	Y	Y	Y	Y	Y
Use of uniform classification systems (i.e. standardised classification system)	Y	Y	Y	Y	Y	Y
System can be integrated with other data collections/compatible data collections	Y	Y	Y	Y	Y	Y
Legislative requirement for collection of data	Y	Y	Y	Y	Y	Y
Simplicity	Y	Y	Y	Y	Y	Y
Timeliness	Y	Y	Y	Y	Y	Y
Flexibility	Y	Y	Y	Y	Y	Y
Quality control measures	Y	Y	Y	Y	Y	Y
Data confidentiality	Y	Y	Y	Y	Y	Y
Individual privacy	Y	Y	Y	Y	Y	Y
System security	Y	Y	Y	Y	Y	Y
Stability of the system	Y	Y	N	Y	Y	N
						
**Practical**						
Data accessibility	Y	Y	Y	Y	Y	Y
Acceptability	Y	Y	Y	Y	Y	Y
Usefulness	Y	Y	Y	Y	Y	Y
Data linkage potential	Y	Y	Y	Y	Y	Y
Geocoding potential	Y	Y	Y	Y	Y	Y
Compatible denominator data	Y	Y	N	Y	Y	N
Routine data analysis	Y	Y	Y	Y	Y	Y
Guidance material for data interpretation	Y	Y	Y	Y	Y	Y
Routine dissemination of information	Y	Y	Y	Y	Y	Y
Adequate resources/cost	N	Y	N	Y	Y	N
Communication support	N	N	N	N	N	N
Coordination support	N	N	N	N	N	N
Effectiveness of system in supporting programs	N	N	N	N	N	N
Efficiency of resource use	N	N	N	N	N	N
Portability	N	N	Y	Y	Y	N
Practicality of system	N	N	N	N	N	N
Relevance of data to users	N	N	N	N	N	N
Supervision support function	N	N	N	N	N	N
Training support functions	N	Y	N	Y	N	N

^1'^Y' indicates the characteristic meets SMART criteria and 'N' indicates the characteristic does not meet SMART criteria.

### Stage 2: Review of surveillance system characteristics

The characteristics were grouped into three categories based on the nature of the information they provide on injury surveillance. These were: (1) *data quality characteristics*, which provide evaluative information regarding the quality of the information obtained from a surveillance system; (2) *operational characteristics*, which describe key aspects or processes governing the way a surveillance system works; and (3) *practical characteristics*, which describe the functional capabilities and practical elements of a system. Each characteristic was assessed by two of the authors using the SMART criteria and initial agreement was reached for 80% of characteristics. The remaining characteristics were discussed and final SMART ratings for these characteristics determined. Twenty-eight characteristics were judged to meet all five SMART criteria. This included all characteristics in the data quality group, all but one operational characteristic (ie. stability of the system), and just under half of the practical characteristics (Table 1).

### Stage 3: Assessment of characteristics by expert opinion

#### Modified-Delphi round one

The aim of round one of the modified-Delphi was to review the proposed definitions of eleven characteristics for which the literature review failed to identify any consistent definition. The appropriateness, practicality and importance of each of these characteristics was assessed.

The results for the panel's ratings of the appropriateness of the proposed definitions for the 11 characteristics that had not been consistently defined in the literature are shown in Table 2. For two characteristics, sensitivity and timeliness, the panel reached 100% agreement on the proposed definitions. For most of the remaining definitions the panel rated them as '*very/extremely*' or *'moderately' *appropriate. The definitions of the nine characteristics that did not achieve 100% agreement by the expert panel were revised based on the panel's comments.

**Table 2:** Expert panel rating of the appropriateness of the definition of each characteristic to assess an injury surveillance system (modified-Delphi rounds 1 and 2)

	**Modified-Delphi – round 1**						**Modified-Delphi – round 2**					
**Characteristic^1^**	**Not at all/somewhat** **(n = 7)**		**Moderate** **(n = 7)**		**Very/extremely** **(n = 7)**		**Not at all/somewhat** **(n = 7)**		**Moderate** **(n = 7)**		**Very/extremely** **(n = 7)**	
	**n**	**%**	**n**	**%**	**n**	**%**	**N**	**%**	**n**	**%**	**n**	**%**
**Data quality**												
Data completeness	1	14.3	2	28.6	4	57.1	1	14.3	-	-	6	85.7
Sensitivity ^2^	-	-	-	-	7	100	-	-	-	-	7	100
Specificity	1	14.3	-	-	6	85.7	1	14.3	1	14.3	5	71.5
Positive predictive value	2	28.6	1	14.3	4	57.1		-	-	-	7	100
Representativeness	3	42.9	-	-	4	57.1	-	-	1	14.3	6	85.7
Positive likelihood ratio	3	42.9	1	14.3	3	42.9	-	-	1	14.3	6	85.7
												
**Operational**												
Simplicity	2	28.6	3	42.9	2	28.6	-	-	3	42.9	4	57.2
Timeliness ^2^	-	-	-	-	7	100	-	-	-	-	7	100
Flexibility	2	28.6	2	28.6	4	57.1	-	-	4	57.1	3	42.9
												
**Practical**												
Acceptability	2	28.6	2	28.6	3	42.9	1	14.3	3	42.9	3	42.9
Usefulness	1	14.3	2	28.6	4	57.1	-	-	1	14.3	6	85.7

^1 ^High consensus was considered to be 70% and above agreement, moderate consensus 50% to 69% agreement, and low consensus less than 50% agreement.

^2^ The panel reached 100% agreement on the proposed definition in round one.

The panel's ratings of the practicality of each characteristic varied. Four characteristics, usefulness, simplicity, data completeness, and timeliness were most commonly rated by the panel as '*very/extremely*' practical to assess in an injury surveillance system. Five characteristics, acceptability, sensitivity, specificity, representativeness, and flexibility were most commonly rated as '*moderate*'. The PPV was rated as either '*moderate*' and '*not at all/somewhat*' (both 42.9%) and the LR+ was most commonly rated as '*not at all/somewhat*' practical to assess.

The ratings of the importance of each characteristic showed that almost all characteristics were rated as important. The exceptions were flexibility, which was mainly rated as '*moderate' *(71.4%), and the PPV and LR+ which were rated by the panel as '*not at all' *or *'somewhat important' *(both 42.9%).

#### Modified-Delphi round two

The aim of the second Delphi exercise was to review the definitions modified after the first round and to obtain ratings of the importance of all characteristics that remained after the SMART criteria assessment. To evaluate the appropriateness of the revised definitions, ratings of the revised definitions were compared with the ratings of the original definitions used in round one. Ratings improved for almost all characteristics. All data quality characteristics, one operational (i.e. timeliness) and one practical (i.e. usefulness) characteristic were rated by the majority of the panel as '*very/extremely*' appropriate in round 2 (Table 2).

Ratings of the importance of the six data quality characteristics showed high consensus and high scores for all characteristics, except LR+ (Table 3). Ratings of the importance of the 14 operational characteristics showed high scores and consensus for nine characteristics. Three characteristics had low mean scores (i.e. simplicity, flexibility and system integration) and two were rated inconsistently low (i.e. legislative requirement for collection of data and data adequacy for injury surveillance) (Table 3). Ratings of the importance of the eight practical characteristics showed high mean ratings and consensus for all characteristics, except potential for data linkage, potential for geocoding and routine dissemination of information (Table 3).

**Table 3:** Expert panel rating of the importance of each characteristic to assess either the data quality, operation or practical ability of an injury surveillance system (modified-Delphi round 2)

**Characteristic**	**Mean^1^** **(n = 7)**	**Median^2^** **(n = 7)**	**Standard Deviation** **(n = 7)**	**Interquartile Range** **(n = 7)**	**Consensus^3^**
**Data quality characteristics**					
Data completeness	6.3	6.0	0.8	1	High
Sensitivity	6.1	6.0	0.9	2	High
Specificity	5.9	6.0	0.9	2	High
Positive predictive value	5.9	6.0	0.9	2	High
Representativeness	6.4	6.0	0.5	1	High
Positive likelihood ratio	5.0	5.0	1.9	4	Moderate
**Operational characteristics**					
Clear purpose and objective(s)	6.6	7.0	0.5	1	High
Data collection process	6.3	6.0	0.5	1	High
Clear case definition	6.7	7.0	0.8	0	High
Legislative requirement for collection of data	4.4	5.0	2.2	3	Low
Type of data collected is adequate for injury surveillance	5.6	6.0	2.5	1	Low
Simplicity	5.4	6.0	0.8	1	High
Timeliness	6.1	6.0	0.4	0	High
Flexibility	5.3	5.0	0.8	1	High
Quality control measures	6.6	7.0	0.5	1	High
Data confidentiality	6.3	6.0	0.8	1	High
Individual privacy	6.3	6.0	0.5	1	High
System security	6.9	7.0	0.4	0	High
Use of uniform classification systems	6.3	6.0	0.5	1	High
System can be integrated with other data collections	5.6	6.0	1.0	1	High
**Practical characteristics**					
Data accessibility	6.4	7.0	0.8	1	High
Potential for data linkage	5.6	6.0	1.1	2	Moderate
Potential for geocoding	5.0	5.0	0.8	2	High
Routine data analysis	6.4	6.0	0.5	1	High
Guidance material for data interpretation	6.1	6.0	0.7	1	High
Routine dissemination of information	6.1	6.0	1.1	1	Moderate
Acceptability	6.3	6.0	0.5	1	High
Usefulness	6.7	7.0	0.5	1	High

^1 ^Mean rating score using seven-point Likert scale (7 represents extremely important).

^2 ^Median rating score using seven-point Likert scale (7 represents extremely important).

^3 ^High consensus was considered to be 1 SD away from the mean, moderate consensus between 1 and 2 SDs away from the mean, and low consensus between 2 and 3 SDs away from the mean.

At the conclusion of the modified-Delphi study, the definitions of six data quality, two operational and one practical characteristic of an injury surveillance system were all rated as appropriate and were considered suitable for use in an EFISS (see Table 4). The definitions of flexibility and acceptability were not rated as '*very/extremely' *appropriate by the majority of experts and were, therefore, not considered suitable for use in an EFISS and were removed from the framework. Tables 5, 6 and 7 show the final definitions for each included characteristic.

**Table 4:** Recommended characteristics for inclusion in an evaluation framework for injury surveillance systems

	**EFISS characteristics**
**Data quality characteristics**	Five characteristics were identified to assess the data quality of an injury surveillance system, including: data completeness, sensitivity, specificity, positive predictive value, and representativeness.
**Operational characteristics**	Nine characteristics were identified to assess the operation of an injury surveillance system, including: system purpose and objectives, data collection process, case definitions, timeliness, quality control measures, data confidentiality, individual privacy, system security, and uniform classification systems.
**Practical characteristics**	Four characteristics were identified to assess the practical capability of an injury surveillance system, including: data accessibility, routine data analysis, guidance material to aid interpretation, and usefulness.

**Table 5:** Rating criteria for the data quality characteristics of the evaluation framework for injury surveillance systems

**Data quality characteristics**	**EFISS definition^1^**		**Rating criteria**
Data completeness	Data completeness will refer to an assessment of the proportion of: (i) missing; (ii) 'not known'; (iii) 'other specified'; and (iv) 'unspecified' data recorded for key characteristics of the injured population (i.e. WHO's core minimum data set for injury surveillance).	I	There is no missing, not known, other specified or unspecified data and this is considered to be *very high*.
		II-1	The proportion of missing, not known, other specified or unspecified data is less than 5% and this is considered to be *high*.
		II-2	The proportion of missing, not known, other specified or unspecified data is less than 15% and this is considered to be *high*.
		II-3	The proportion of missing, not known, other specified or unspecified data is less than 25% and this is considered to be *high*.
		III	The proportion of missing, not known, other specified or unspecified data is in the range 26 to 50% and this is considered to be *low*.
		IV	The proportion of missing, not known, other specified or unspecified data is in the range 51 to 100% and this is considered to be *very low*.

Sensitivity	Sensitivity will refer to the ability to correctly detect all cases of true injury events that the data collection intended to detect in the target population.	I	Sensitivity is in the range 90 to 100% and is considered to be *very high*.
		II	Sensitivity is in the range 71 to 89% and is considered to be *high*.
		III	Sensitivity is in the range 51 to 70% and is considered to be *low*.
		IV	Sensitivity is less than 50% and is considered to be *very low*.

Specificity	Specificity will refer to the ability to correctly detect all non-injury cases that the data collection should not have detected as injury cases in the target population	I	Specificity is in the range 90 to 100% and is considered to be *very high*.
		II	Specificity is in the range 71 to 89% and is considered to be *high*.
		III	Specificity is in the range 51 to 70% and is considered to be *low*.
		IV	Specificity is less than 50% and is considered to be *very low*.

Positive predictive value	The PPV will refer to the number of correctly identified true injury cases divided by the total number of cases that are identified (correctly and incorrectly) as an injury case from the target population.	I	PPV is in the range 90 to 100% and is considered to be *very high*.
		II	PPV is in the range 71 to 89% and is considered to be *high*.
		III	PPV is in the range 51 to 70% and is considered to be *low*.
		IV	PPV is less than 50% and is considered to be *very low*.

Representative-ness	Representativeness will refer to the ability of the collection to provide an accurate representation of the distribution of key characteristics of the injured population (i.e. WHO's core minimum data set for injury surveillance) in a sample of the target population.	I	Appropriate statistical tests (e.g. Chi squared test, Fisher's Exact test) confirm there is no significant difference in the distribution of key characteristics of the injured population^1 ^between data in the surveillance system being evaluated to a gold standard (or other) data collection and the data is considered representative of the target population.
		IV	Appropriate statistical tests confirm there is a significant difference in the distribution of key characteristics of the injured population^1 ^between data in the surveillance system being evaluated to a gold standard (or other) data collection and the data is not considered representative of the target population.

^1 ^WHO's core minimum data set for injury surveillance includes information regarding individual demographics (i.e. age, sex), the circumstances of the injury event (i.e. intent, activity, place of occurrence, mechanism of injury), and the injury outcome (i.e. nature of injury).

**Table 6:** Rating criteria for the operational characteristics of the evaluation framework for injury surveillance systems

**Operational characteristics**	**EFISS definition^1^**		**Rating criteria**
Purpose and objectives	The purpose of the injury surveillance system, the reason why the system exists, and objectives of the injury surveillance system, what the information from the system is used for, should be described.	I	If the purpose and/or objectives of the data collection include injury surveillance, it rates as *very high*.
		II	If the purpose and/or objectives of the data collection include monitoring of trends or conducting research, it rates as *high*.
		III	If the purpose and/or objectives of the data collection include other rationales, such as resource allocation or planning, it rates as *low*.
		IV	If the purpose and/or objectives of the data collection are not stated, it rates as *very low*.

Data collection process	The method of data collection for an injury surveillance system and the number of steps involved in data collection should be examined using a data collection flow chart.	I	If the data collection process takes one to three steps to complete, it rates as *very high*.
		II	If the data collection process takes four to six steps to complete, it rates as *high*.
		III	If the data collection process takes seven to nine steps to complete, it rates as *low*.
		IV	If the data collection process takes ten or more steps to complete, it rates as *very low*.

Case definition	The injury case definition adopted by an injury surveillance system to identify cases should be described.	I	If variables in the data collection can identify the injury cases of interest it rates as *very high*.
		IV	If variables in the data collection can not identify injury cases of interest it rates as *very low*.

Timeliness	Timeliness will refer to the time taken to accomplish each of the three surveillance phases of: (i) data collection; (ii) data analysis and interpretation; and (iii) dissemination.	I	If the time taken to complete data collection, data analysis, interpretation and dissemination is daily to monthly, it rates as *very high*.
		II	If the time taken to complete data collection, data analysis, interpretation and dissemination is annual to biennial, it rates as *high*.
		III	If the time taken to complete data collection, data analysis, interpretation and dissemination is greater than biennial, it rates as *low*
		IV	If data is not either routinely collected, analysed, interpreted or disseminated, it rates as *very low*.

Uniform classification systems	The classification system(s) used to record information in the injury surveillance system for variables in the WHO's core minimum and optimal data sets for injury surveillance should be identified.	I	If standard classification systems are used to record information for 76 to 100% of variables in the core minimum and optional data sets for injury surveillance, it rates as *very high*.
		II	If standard classification systems are used to record information for 51 to 75% of variables in the core minimum and optional data sets for injury surveillance, it rates as *high*.
		III	If standard classification systems are used to record information for 26 to 50% of variables in the core minimum and optional data sets for injury surveillance, it rates as *low*.
		IV	If standard classification systems are not used or are used to record information for less than 25% of variables in the core minimum and optional data sets for injury surveillance, it rates as *very low*.

Quality control measures	The quality control measures regularly utilised by the agency responsible for the injury surveillance system should be identified.	I	If quality control measures are in place and are conducted, it rates as *very high*.
		IV	If there are no quality control measures in place, it rates as *very low*.

Confidentiality and privacy	The methods by which an individual's information in the injury surveillance system is safe guarded against disclosure should be described.	I	If data users are required to sign a confidentiality and/or data security agreement, it rates as *very high*.
		IV	If data users are not required to sign a confidentiality and/or data security agreement, it rates as *very low*.

System security	The data access requirements (e.g. password protection) that safe guard against the disclosure of confidential information should be described.	I	If there are data access procedures in place (e.g. password protection) to safe guard against the disclosure of confidential information, it rates as *very high*.
		IV	If there are no data access procedures in place to safe guard against the disclosure of confidential information, it rates as *very low*.

^1 ^WHO's core minimum data set for injury surveillance includes information regarding individual demographics (i.e. age, sex), the circumstances of the injury event (i.e. intent, activity, place of occurrence, mechanism of injury), and the injury outcome (i.e. nature of injury).

**Table 7:** Rating criteria for the practical characteristics of the evaluation framework for injury surveillance systems

**Practical characteristics**	**EFISS definition^1^**		**Rating criteria**
Data accessibility	The method by which potential data users access data from the injury surveillance system should be reported.	I	If data is accessible for data users in unit record format from an internet-based interface and/or data warehouse (or similar), it rates as *very high*.
		II	If data is accessible for data users in unit record format from a CD-ROM (or other data storage device), it rates as *high*.
		III	If data is accessible for data users in an aggregate format only, it rates as *low*.
		IV	If data is not accessible by data users, it rates as *very low*.

Usefulness	Usefulness will refer to the ability to contribute to the identification of potential key areas for preventive action in terms of the ability to: (a) identify new and/or emerging injury mechanisms; (b) monitor injury trends over time; and (c) describe key characteristics of the injured population (i.e. WHO's core minimum data set for injury surveillance).	I	If the data collection contains 76 to 100% of variables in the core minimum and optional data sets for injury surveillance, it rates as *very high*.
		II	If the data collection contains 51 to 75% of variables in the core minimum and optional data sets for injury surveillance, it rates as *high*.
		III	If the data collection contains 26 to 50% of variables in the core minimum and optional data sets for injury surveillance, it rates as *low*.
		IV	If the data collection contains less than 25% of variables in the core minimum and optional data sets for injury surveillance, it rates as *very low*.

Data analysis	The routine data analyses conducted using data from the injury surveillance system by the agency responsible for the surveillance system should be described.	I	If data analysis is conducted daily to monthly or on request and results of this analysis are available for all data users, it rates as *very high*.
		II	If data analysis is conducted annually to biennially and results of this analysis are available for all data users, it rates as *high*.
		III	If data analysis is conducted greater than biennially and results of this analysis are available for all data users, it rates as *low*.
		IV	If data analysis is not conducted, it rates as *very low*.

Guidance material to aid data interpretation	The availability of guidance material on the interpretation of data from the injury surveillance system should be described.	I	If there is an up-to-date data dictionary, manual or data user's guide and routine contact with data users regarding data analysis issues to aid data interpretation, it rates as *very high*.
		II	If there is an up-to-date data dictionary, manual or data user's guide to aid data interpretation, it rates as *high*.
		III	If there is a data dictionary, manual or data user's guide to aid data interpretation, but this documentation in not kept up-to-date, it rates as *low*.
		IV	If there is no documentation or guidance material to aid data interpretation, it rates as *very low*.

^1 ^WHO's core minimum data set for injury surveillance includes information regarding individual demographics (i.e. age, sex), the circumstances of the injury event (i.e. intent, activity, place of occurrence, mechanism of injury), and the injury outcome (i.e. nature of injury).

### Stage 4: Development of a rating system for the EFISS characteristics

The framework adopted to create the rating scales for each EFISS characteristic was the same framework used by the evidenced-based medicine (EMB) field [27,28]. This framework was chosen as the hierarchical structure of this framework and its use of clearly defined rating criteria have been successfully applied in other areas, such as public health interventions [53].

There was only minimal guidance from the literature regarding what might be considered to represent either *high *or *low *ratings of each EFISS characteristic. For example, Hasbrouck et al [54] believed that the sensitivity of the detection of violent injuries in Kingston, Jamaica which ranged from 62% to 69% were '*adequate*' and that a PPV of 86% was '*high*'. Similarly, Hedegaard et al [55] stated that a PPV of 89% was '*high*' in the confirmation of firearm-related injuries in Colorado, while Wiersema et al [56] reported a PPV of 99.6% to be '*very high*' and referred to a sensitivity of 99.6% as '*extremely sensitive*' in the detection of firearm-related injuries in Maryland. At the other end of the spectrum, McClure and Burnside [57] considered the sensitivity of the detection of injuries in the Australian Capital Territory Injury Surveillance and Prevention Project at 31% to be '*low*'.

The rating scales developed for the EFISS are based on the (limited) previous research and the authors' professional judgment. A four-level rating scheme is proposed for most characteristics, composed of I '*very high', II 'high', III 'low', and *IV '*very low'*. For five characteristics a dichotomous scale is proposed using I and IV. These are set out in Tables 5, 6 and 7.

## Discussion

Injury surveillance systems have lacked a systematic framework by which they could be evaluated. Evaluation frameworks exist for other public health surveillance systems [8-11], but none of these are specific to an injury surveillance system, and their methods of development are generally poorly described. This paper describes the development of an evaluation framework specifically designed to evaluate an injury surveillance system. It used a systematic process involving several rounds of review and analysis and has resulted in 18 characteristics. The strengths of this new framework are that the characteristics included have been tested using relevant subject matter experts in terms of their clarity of definition and their importance for evaluating injury data collections. The new framework could be applied to any type of injury data.

The process revealed considerable disagreement among experts as to the meaning and relevance of some of the potential characteristics, but high levels of agreement for others. In general those characteristics that were rated low in importance, or where there was considerable dispute about their importance, were excluded. One characteristic, acceptability, was also excluded because of disagreement about its definition despite being rated high in importance. This highlights the problem of using loosely defined characteristics in evaluations. Future refinement of this framework may consider incorporating this characteristic in some way. The remaining 18 characteristics have the advantages of clear definitions and relatively high-rated importance.

It could be argued that the standards adopted for including characteristics in the EFISS were too high and that some additional characteristics should be included. Indeed the core set of characteristics could be enlarged to an optional additional set to include all or some of the ten characteristics that had been previously excluded. This would involve one additional data quality (i.e. LR+), five additional operational (i.e. legislative requirement for data collection, data adequacy for injury surveillance, simplicity, flexibility, and system integration) and four additional practical (i.e. acceptability, potential for data linkage and geocoding, and routine dissemination) characteristics. However, the definitions of some of these (e.g. flexibility and acceptability) would need further refinement, and a number of these characteristics were rated as low in importance and had little consistency between raters (e.g. legislative requirement for data collection, and adequacy of data for injury surveillance). While it is certainly true that the characteristics employed in an evaluation can vary with the purpose of the evaluation, poorly defined characteristics that result in inconsistent ratings between raters will never be useful. Furthermore, there is a core set of characteristics of any data collection that form the basis for its use no matter what the purpose, data completeness and clear case definition, for example. As the purpose for evaluation of injury data was not specified for the expert raters, it is not surprising that these core characteristics emerged as the most important.

The EFISS includes a rating system for assessing the adequacy of each characteristic, the first such attempt for a public health-related surveillance system [8-10,41,58]. Further work may ultimately lead to refinement of the rating system, although the most appropriate rating criteria will likely vary with context.

There are several strengths of the current study. First, it adopted a broad literature search strategy to include reports prepared by government and non-government organisations as well as academia. This captured the broadest range of the existing evaluation frameworks since many were not published in the peer-reviewed literature. Second, the study used generally accepted criteria (SMART) as well as expert judgment for testing potential evaluation characteristics [8-11]. Lastly the study took a systematic, a priori approach to defining consensus during the modified-Delphi study through a technique of specifying a consensus range (ie. high, moderate, low) [26].

It is arguable that the results of this research may have been influenced by the nature of the Delphi panel. Even though the selection of panel members attempted to include all of the major experts in injury surveillance in Australia, the panel members self-selected to an extent as not all responded and three declined due to understandable reasons of other commitments. There were no obvious differences in the characteristics (age, experience, work context) between participants and non-participants. Furthermore, while all participants were from Australia, many of the participants had worked with international data collections and so are familiar with a range of types of injury data collections and with the different purposes to which they could be put. Whether the results would change with a larger group of injury surveillance experts working in another country remains to be established. The expert panel did consist of only seven members and while there are no strict rules governing the number of Delphi panel members [59] this low number was not ideal as the opinion of one or two experts could notably alter results. There is little or no agreement regarding the appropriate size of a expert panel for use in a Delphi study [59-61]. Mitchell [62] states that a panel should have at least eight to ten members, but may be as large or as small as resources and time allow. On the other hand, Brockhoff [63] considers that five to nine participants can perform well using the Delphi process. Therefore, the number of panelists in the current study is not outside the limits of what is considered appropriate, or practical, to contribute to a Delphi study. Furthermore, although some Delphi studies use multiple rounds of review, only two rounds were used in this study to reduce the likelihood of questionnaire fatigue on participants [64,65]. However it is possible that additional Delphi rounds may have resulted in more characteristics being included in the EFISS, as further revision of characteristic definitions may have resulted in higher ratings of appropriateness and importance by the expert panel.

## Conclusion

The EFISS has built upon existing evaluation frameworks for surveillance systems to produce a framework to guide the evaluation of an injury surveillance system. It is offered as a prototype evaluation framework that has clear developmental foundations. While it can be used in its current form, it could certainly be developed further. For example, the EFISS could include a weighting system to adjust for the importance of different EFISS characteristics. In addition, the interrelationships between characteristics may also be considered within the rating system. Further testing may result in more precise and hence more useful definitions of problem characteristics like acceptability. In the meantime, the EFISS is offered to assist agencies operating injury surveillance systems to identify areas for data quality and system improvement.

## Competing interests

The authors declare that they have no competing interests.

## Authors' contributions

RM conducted the literature reviews and Delphi study. RM and AW assessed the characteristics using the SMART criteria. RM prepared the first draft of the manuscript. All authors contributed to the development of ideas expressed in the manuscript and assisted in preparing the final version. All authors read and approved the final manuscript.

## Pre-publication history

The pre-publication history for this paper can be accessed here:


